# Prevalence and Associated Workplace Factors of Musculoskeletal Discomfort Among Critical Care Nurses in a Malaysian Teaching Hospital

**DOI:** 10.3390/healthcare14142134

**Published:** 2026-07-16

**Authors:** Nik Nur Iwana Iznin Nik Din, Mohd Ismail Ibrahim, Mohd Shaharudin Shah Che Hamzah, Anis Kausar Ghazali

**Affiliations:** 1 Department of Community Medicine, School of Medical Sciences, Universiti Sains Malaysia, Kubang Kerian 16150, Kelantan, Malaysia; iwana@usm.my; 2 Department of Emergency Medicine, Hospital Pakar Universiti Sains Malaysia, Kubang Kerian 16150, Kelantan, Malaysia; shaharudin@usm.my; 3 Unit of Biostatistics and Research Methodology, School of Medical Sciences, Universiti Sains Malaysia, Kota Bharu 16150, Kelantan, Malaysia; anisyo@usm.my

**Keywords:** musculoskeletal discomfort, critical care nurses, occupational health, patient handling, nursing workforce, Malaysia

## Abstract

**Highlights:**

**What are the main findings?**

- A high prevalence of musculoskeletal discomfort was identified among critical care nurses, with more than four-fifths reporting severe discomfort.
- Manual orthopedic patient handling and insufficient rest breaks during shifts were significant predictors of severe musculoskeletal discomfort.

**What are the implications of the main findings?**

- Improving ergonomic practices and safe patient handling strategies may reduce the burden of musculoskeletal discomfort among critical care nurses.
- Hospital administrators should strengthen occupational health policies, staffing support, and work-rest scheduling to protect nurses’ well-being and maintain quality patient care.

**Abstract:**

**Background/Objectives**: Musculoskeletal discomfort disorders (MSDs) are common occupational health problems among nurses, particularly in critical care settings due to physically demanding tasks and prolonged patient care activities. However, evidence regarding MSDs among critical care nurses in Malaysian teaching hospitals remains limited. This study aimed to determine the prevalence, severity, and associated workplace factors of musculoskeletal discomfort among critical care nurses in Kelantan, Malaysia. **Methods**: An analytical cross-sectional study was conducted among 308 critical care nurses working at Hospital Pakar Universiti Sains Malaysia, Kelantan. Data were collected using a structured self-administered questionnaire and the Cornell Musculoskeletal Discomfort Questionnaire (CMDQ). Descriptive statistics, simple logistic regression, and multiple logistic regression analyses were performed using IBM SPSS version 30. **Results**: The prevalence of severe musculoskeletal discomfort among respondents was high, with 82.1% reporting severe discomfort. Several workplace-related factors were significantly associated with severe musculoskeletal discomfort in the bivariable analysis, including awkward back bending, carrying heavy equipment, manual orthopedic patient handling, insufficient rest breaks, awkward working positions, working while injured, and inadequate training on injury prevention. Multiple logistic regression analysis identified manual orthopedic patient handling (AOR = 1.882, 95% CI: 1.033–3.426, *p* = 0.039) and insufficient rest breaks during shifts (AOR = 2.581, 95% CI: 1.466–4.543, *p* = 0.001) as significant predictors of severe musculoskeletal discomfort. **Conclusions**: Musculoskeletal discomfort is highly prevalent among critical care nurses. Workplace ergonomic strain and inadequate recovery periods significantly contribute to severe musculoskeletal discomfort. Strengthening ergonomic practices, safe patient handling strategies, and occupational health interventions is essential to improve nurses’ well-being and maintain quality patient care.

## 1. Introduction

Nurses play an essential role in healthcare delivery and constitute one of the largest groups of healthcare professionals worldwide. Among them, critical care nurses work in particularly demanding clinical environments characterized by the management of critically ill patients, emergency interventions, advanced life support procedures, and continuous patient monitoring [1,2]. The nature of critical care nursing often requires repetitive patient handling, prolonged standing, awkward postures, frequent bending and twisting movements, lifting tasks, and the mobilization of highly dependent patients [3,4]. These occupational demands place nurses at an increased risk of developing musculoskeletal discomfort disorders (MSDs), which remain a major occupational health concern within healthcare settings.

Musculoskeletal discomfort disorders encompass pain, discomfort, or injuries affecting muscles, tendons, ligaments, joints, and supporting soft tissues resulting from repetitive strain, excessive physical workload, or exposure to ergonomic hazards [5]. MSDs are among the most prevalent work-related health problems affecting nurses globally and are associated with absenteeism, reduced productivity, impaired job performance, diminished quality of life, and increased healthcare expenditure [6,7]. Previous studies have reported that the prevalence of MSDs among nurses ranges from 33% to 88%, with the lower back, neck, shoulders, and knees consistently identified as the most frequently affected anatomical regions [8,9,10]. Beyond their impact on individual health, MSDs may compromise workforce sustainability and potentially affect the quality and safety of patient care.

Critical care nurses are particularly vulnerable to musculoskeletal discomfort due to the physical and ergonomic demands inherent in their daily clinical practice. Critically ill patients frequently require full assistance during repositioning, transfer, mobilization, and hygiene-related procedures, resulting in substantial biomechanical loading on healthcare workers [11]. In addition, critical care units often operate under conditions of high patient acuity, staffing shortages, and increased workload demands, which may contribute to physical fatigue and inadequate recovery time between tasks [12,13]. Previous studies have also highlighted the influence of psychosocial stressors, rotating shift schedules, prolonged working hours, and occupational fatigue on the development and progression of musculoskeletal symptoms among nurses [14].

International evidence consistently demonstrates a high burden of MSDs among nurses working in intensive care and emergency settings. Studies conducted in Egypt, Vietnam, Estonia, Turkey, and Nigeria have reported substantial rates of work-related musculoskeletal disorders and identified occupational factors such as patient handling, repetitive movements, and poor ergonomic conditions as major contributors [9,15,16,17,18]. Similarly, musculoskeletal discomfort has emerged as an important occupational health issue among Malaysian nurses. A recent Malaysian scoping review reported that the prevalence of low back pain among nurses reached as high as 67.9% [19], while another local study found that 73.1% of nurses experienced musculoskeletal discomfort involving at least one anatomical body region [20]. These findings indicate that MSDs continue to represent a significant challenge within the nursing workforce.

Despite the growing body of evidence, several important knowledge gaps remain. First, most available studies have focused on general nursing populations or included nurses from multiple hospital departments, with relatively limited evidence specifically examining critical care nurses in Malaysian teaching hospitals. Given the unique ergonomic demands, workload intensity, and patient dependency characteristics encountered in critical care settings, findings from broader nursing populations may not adequately reflect the experiences of critical care nurses.

Second, although previous studies have reported overall prevalence estimates of musculoskeletal disorders among nurses, relatively little attention has been given to the anatomical distribution and severity of musculoskeletal discomfort among critical care nurses in Malaysian healthcare settings. Understanding which anatomical body regions are most frequently affected and the extent of discomfort experienced in these regions is important for identifying occupation-specific patterns of musculoskeletal strain and prioritizing targeted ergonomic interventions. Furthermore, evidence regarding workplace factors associated with severe musculoskeletal discomfort among critical care nurses remains insufficient, particularly in teaching hospital settings where patient complexity and workload demands may differ from other healthcare institutions.

Addressing these knowledge gaps is important because musculoskeletal discomfort may adversely affect nurses’ physical health, work performance, job satisfaction, retention, and ultimately patient care quality and safety [21,22]. A better understanding of the prevalence, anatomical distribution, severity, and workplace determinants of musculoskeletal discomfort among critical care nurses may assist healthcare institutions in designing evidence-based ergonomic interventions, strengthening safe patient handling programmes, and implementing occupational health policies that support a healthier and more sustainable nursing workforce.

Therefore, this study aimed to determine the prevalence, anatomical distribution, and severity of musculoskeletal discomfort and to identify associated workplace factors among critical care nurses working in Hospital Pakar Universiti Sains Malaysia, Kelantan.

## 2. Materials and Methods

### 2.1. Study Design and Setting

An analytical cross-sectional study was conducted among critical care nurses working at Hospital Pakar Universiti Sains Malaysia (Hospital Pakar USM), Kelantan, Malaysia. Hospital Pakar USM is a tertiary teaching hospital that provides specialized healthcare services and intensive care management for critically ill patients.

### 2.2. Study Population and Sampling

The study population consisted of registered nurses working in critical care units at Hospital Pakar Universiti Sains Malaysia, including the Intensive Care Unit (ICU), Coronary Care Unit (CCU), Trauma Intensive Care Unit, Neonatal Intensive Care Unit (NICU), High Dependency Units, and Emergency Critical Care areas. Nurses who were directly involved in patient care and had worked in critical care settings for at least six months were eligible for inclusion.

Nurses who were on long-term medical leave, maternity leave, administrative placement, or extended absence during the data collection period were excluded from the study. The required sample size was calculated using a single population proportion formula based on the estimated prevalence of musculoskeletal discomfort reported among Malaysian nurses [20]. After adjustment for potential non-response, the minimum required sample size was 308 participants.

A proportionate random sampling method was employed. A complete list of critical care nurses from each participating unit was obtained from the respective head nurses. The sampling frame was subsequently screened according to the study eligibility criteria, and participants were selected proportionately from each unit using random sampling procedures to ensure adequate representation across all critical care settings.

The total eligible population consisted of 419 critical care nurses. All selected nurses were invited to participate, and completed questionnaires were included in the final analysis.

### 2.3. Study Instrument

Data were collected using a structured self-administered questionnaire comprising three sections.

The first section collected respondents’ sociodemographic and occupational information, including age, sex, marital status, educational level, duration of employment, and working unit. These variables were included to characterize the study population and to explore their potential association with musculoskeletal discomfort.

The second section assessed workplace-related ergonomic and occupational factors potentially associated with musculoskeletal discomfort among critical care nurses. The items consisted of dichotomous (Yes/No) questions that evaluated exposure to common ergonomic risk factors reported in previous studies of work-related musculoskeletal disorders among nurses and healthcare workers [23,24,25,26]. These factors included prolonged standing, repetitive movements, awkward working postures, bending or twisting of the back, manual patient handling, lifting and transferring patients, carrying heavy equipment, inadequate rest breaks during shifts, working while injured, reaching away from the body, and inadequate training on injury prevention. These items were designed to identify the presence or absence of specific workplace exposures rather than to measure an underlying latent construct. Therefore, psychometric reliability testing was not performed. Nevertheless, the questionnaire items were adapted from previously published literature and reviewed by the research team to ensure clarity, relevance, and suitability for the critical care nursing environment [23,24,25,26].

The third section utilized the Cornell Musculoskeletal Discomfort Questionnaire (CMDQ), a widely used ergonomic assessment instrument designed to evaluate work-related musculoskeletal discomfort across multiple anatomical regions [27]. The CMDQ assesses discomfort in the neck, shoulders, upper back, lower back, upper arms, forearms, wrists/hands, hips/thighs, knees, lower legs, and feet. Respondents were asked to report musculoskeletal discomfort experienced during the previous seven days for each anatomical region assessed.

The CMDQ evaluates three dimensions of musculoskeletal discomfort: frequency of discomfort, discomfort intensity, and the extent to which the discomfort interferes with work activities. Frequency was scored as 0 (never), 1.5 (1–2 times during the previous week), 3.5 (3–4 times during the previous week), 5 (once every day), and 10 (several times every day). Discomfort intensity was scored as 1 (slightly uncomfortable), 2 (moderately uncomfortable), and 3 (very uncomfortable). Work interference was scored as 1 (not at all), 2 (slightly interfered), and 3 (substantially interfered). The musculoskeletal discomfort score for each anatomical region was calculated using the standard CMDQ frequency–discomfort–interference (FDI) formula:

Musculoskeletal Discomfort Score = Frequency × Discomfort × Interference

Based on the CMDQ scoring classification, discomfort scores were categorized as no discomfort (score = 0), mild discomfort (score = 1.5), moderate discomfort (score = 1.6–10.5), and severe discomfort (score > 10.5). For the purpose of logistic regression analysis, respondents were subsequently classified into two categories: mild-to-moderate musculoskeletal discomfort and severe musculoskeletal discomfort. This categorization was performed to facilitate the identification of workplace factors associated with severe musculoskeletal discomfort among critical care nurses.

The CMDQ has been widely used in occupational health and ergonomics research to assess both the severity and functional impact of musculoskeletal discomfort and is considered an appropriate instrument for evaluating work-related musculoskeletal symptoms among healthcare workers [27].

### 2.4. Data Collection Procedure

Data collection was conducted between March and June 2025 after obtaining ethical approval from the Human Research Ethics Committee of Universiti Sains Malaysia and permission from the hospital administration. Prior to data collection, approval was obtained from the respective nursing managers and head nurses of all participating critical care units.

A list of nurses working in each critical care unit was obtained from the respective head nurses. The sampling frame was screened according to the study inclusion and exclusion criteria. Subsequently, participants were selected using proportionate random sampling to ensure representation from all critical care units.

Selected nurses were approached individually by the researcher during scheduled visits to the respective units. The study objectives, procedures, potential benefits, and participants’ rights were explained verbally and through a participant information sheet. Nurses were informed that participation was entirely voluntary and that they could withdraw from the study at any stage without any consequences to their employment or professional responsibilities. Written informed consent was obtained prior to participation.

Participants were provided with a self-administered questionnaire package consisting of the sociodemographic and occupational characteristics questionnaire, workplace ergonomic factors questionnaire, and the Cornell Musculoskeletal Discomfort Questionnaire (CMDQ). Respondents completed the questionnaires independently during their available time either before, during, or after their work shifts. The average time required to complete the questionnaire was approximately 15–20 min.

To maintain confidentiality and anonymity, no personal identifiers were collected. Completed questionnaires were returned in sealed envelopes and deposited into designated collection boxes located within the participating units. The researcher subsequently collected the completed questionnaires and reviewed them for completeness. All 308 selected nurses completed the questionnaire, resulting in a response rate of 100%. No questionnaires contained missing data and all responses were included in the final analysis.

### 2.5. Statistical Analysis

Data were analyzed using IBM SPSS Statistics version 30 (IBM Corp., Armonk, NY, USA). Descriptive statistics were used to summarize socio-demographic characteristics and prevalence of musculoskeletal discomfort. Categorical variables were presented as frequencies and percentages, whereas continuous variables were summarized using means and standard deviations.

Simple logistic regression analysis was performed to determine factors associated with severe musculoskeletal discomfort. Variables with *p*-values less than 0.25 in the bivariable analysis were included in the multiple logistic regression model to avoid excluding potentially important predictors during preliminary screening, as recommended by Hosmer and Lemeshow [28]. Adjusted odds ratios (AORs) with 95% confidence intervals were reported. Statistical significance was set at *p* < 0.05.

### 2.6. Ethical Considerations

The study was conducted in accordance with the Declaration of Helsinki and approved by the Human Research Ethics Committee of Universiti Sains Malaysia (USM/JEPeM/KK/24030264). Participation was voluntary, and informed consent was obtained from all respondents prior to data collection.

## 3. Results

### 3.1. Sociodemographic of Respondents

Table 1 presents the sociodemographic characteristics of the 308 critical care nurses included in this study. Most respondents were aged between 31 and 40 years (68.2%), were female (88.0%), and married (85.4%). Nearly all respondents held a diploma in nursing (97.1%). The mean body weight, height, and BMI were 66.51 (SD = 15.48) kg, 1.61 (SD = 0.84) m, and 26.88 (SD = 5.54) kg/m^2^, respectively. These variables were collected to describe the study population, while the subsequent multivariable analysis focused specifically on workplace-related factors in accordance with the study objective.

Table 2 describes the working characteristics and workplace-related factors associated with musculoskeletal discomfort among critical care nurses. The highest proportion of respondents worked in the Emergency and Trauma Unit (20.8%) and Neonatal Intensive Care Unit (14.0%). Most nurses had between 6 and 10 years of service (28.6%). A large proportion of respondents reported exposure to physically demanding tasks and ergonomic risk factors, including lifting or transferring dependent patients (89.3%), prolonged standing or maintaining the same position (73.7%), assisting patients during gait activities (68.2%), treating excessive numbers of patients per day (62.3%), and performing repetitive tasks (58.8%). Additionally, half of the respondents reported inadequate rest breaks during work shifts (50.0%). The descriptive findings indicate that a substantial proportion of critical care nurses reported exposure to physically demanding and ergonomically challenging workplace conditions.

### 3.2. Prevalence of Musculoskeletal Discomfort Among Respondents

A total of 308 completed questionnaires were included in the final analysis. The prevalence of musculoskeletal discomfort among critical care nurses was high. Overall, 250 respondents (82.1%) reported severe musculoskeletal discomfort, while 55 respondents (17.9%) experienced mild to moderate discomfort.

Table 3 presents the prevalence of musculoskeletal discomfort according to anatomical body region among critical care nurses. The lower back was the most frequently affected body region, with 76.3% of respondents reporting discomfort, followed by the upper back (62.0%), neck (59.1%), right foot (51.0%), and right shoulder (48.7%). In contrast, the lowest prevalence was observed in the left knee (29.9%). These findings indicate that musculoskeletal discomfort predominantly affects body regions subjected to prolonged standing, repetitive movements, and patient handling activities. The table also presents the overall severity classification of musculoskeletal discomfort among respondents.

### 3.3. Factors Associated with Severe Musculoskeletal Discomfort

Table 4 presents the results of the logistic regression analysis examining the association between workplace-related factors and severe musculoskeletal discomfort among critical care nurses. Several occupational factors were found to be significantly associated with severe musculoskeletal discomfort. Nurses who reported bending or twisting their back in an awkward way were 1.8 times more likely to experience severe musculoskeletal discomfort during handling orthopedic patient (COR = 1.788, 95% CI: 1.042–3.068, *p* = 0.035). Similarly, carrying, lifting, or moving heavy materials or equipment (COR = 2.019, 95% CI: 1.173–3.474, *p* = 0.011), performing manual orthopedic techniques (COR = 2.019, 95% CI: 1.123–3.630, *p* = 0.019), and working in awkward or cramped positions (COR = 2.419, 95% CI: 1.240–4.720, *p* = 0.010) were significantly associated with increased odds of severe musculoskeletal discomfort.

In addition, nurses who reported inadequate rest breaks during work shifts were approximately 2.7 times more likely to experience severe musculoskeletal discomfort (COR = 2.734, 95% CI: 1.562–4.786, *p* < 0.001). Other significant factors included continuing to work while injured or hurt (COR = 1.841, 95% CI: 1.064–3.186, *p* = 0.029), reaching away from the body during work activities (COR = 2.255, 95% CI: 1.221–4.162, *p* = 0.009), and inadequate training on injury prevention (COR = 2.113, 95% CI: 1.144–3.904, *p* = 0.017). These findings suggest that ergonomic strain, physically demanding work activities, and insufficient workplace support contribute substantially to severe musculoskeletal discomfort among critical care nurses.

After adjustment for the other workplace-related variables included in the multivariable logistic regression analysis, only two factors remained independently associated with severe musculoskeletal discomfort among critical care nurses. Manual orthopedic patient handling was significantly associated with severe musculoskeletal discomfort (AOR = 1.882, 95% CI: 1.033–3.426, *p* = 0.039). Nurses who frequently performed physically demanding patient handling activities were approximately 1.9 times more likely to experience severe musculoskeletal discomfort than those with lower exposure. Similarly, insufficient rest breaks during work shifts remained a significant independent factor associated with severe musculoskeletal discomfort (AOR = 2.581, 95% CI: 1.466–4.543, *p* = 0.001). Nurses who reported inadequate rest periods during work shifts were approximately 2.6 times more likely to experience severe musculoskeletal discomfort than those who had adequate rest breaks.

## 4. Discussion

This study demonstrated a high prevalence of musculoskeletal discomfort among critical care nurses in a Malaysian teaching hospital, with 82.1% of respondents experiencing severe musculoskeletal discomfort. This finding highlights the substantial occupational health burden faced by nurses working in physically demanding critical care environments and underscores the need for greater attention to workplace ergonomics and occupational health protection within healthcare settings.

The prevalence observed in the present study is generally consistent with previous international and Malaysian studies reporting a high burden of musculoskeletal disorders among nurses [8,9,15,19,20]. However, the proportion of nurses classified as having severe musculoskeletal discomfort in this study was higher than that reported in several previous investigations involving nursing populations [20,23,29,30]. Several factors may explain this observation. First, critical care nurses are routinely exposed to physically demanding activities, including lifting and transferring dependent patients, repositioning critically ill patients, prolonged standing, repetitive tasks, and maintaining awkward postures, all of which were commonly reported by respondents in this study [11,12]. Second, the high-acuity nature of critical care settings often requires continuous patient monitoring and rapid responses to clinical deterioration, which may increase physical workload while limiting opportunities for adequate recovery during work shifts. Third, the CMDQ classification used in this study incorporates not only the presence of symptoms but also the frequency of discomfort, severity of symptoms, and interference with work activities. Consequently, the instrument may provide a more comprehensive assessment of musculoskeletal burden compared with studies that rely solely on symptom prevalence.

The findings also reinforce the importance of workplace ergonomic exposures as major contributors to musculoskeletal discomfort among critical care nurses. More than half of the respondents reported exposure to several recognized ergonomic risk factors, including lifting or transferring dependent patients, prolonged standing, repetitive tasks, awkward body postures, and inadequate rest breaks. These findings are consistent with previous evidence demonstrating that cumulative biomechanical loading and repetitive physical demands substantially increase the risk of musculoskeletal disorders among nurses and other healthcare workers [24,25]. Persistent musculoskeletal discomfort may adversely affect nurses’ physical health, work performance, job satisfaction, and productivity, while potentially compromising the quality and safety of patient care [21,22].

One of the most important findings of this study was the significant association between manual orthopedic patient handling and severe musculoskeletal discomfort. Nurses who performed manual orthopedic patient handling were almost twice as likely to experience severe musculoskeletal discomfort compared with their counterparts. This finding is biologically plausible because critically ill and immobilized patients often require substantial physical assistance during repositioning, transfer, and mobilization procedures. These activities frequently involve lifting, bending, twisting, and sustained force exertion, which increase mechanical stress on the spine, shoulders, and lower extremities [26,31]. Repeated exposure to such activities over time may contribute to cumulative musculoskeletal strain and the development of chronic discomfort.

The association identified in this study is consistent with previous research that has identified manual patient handling as one of the strongest occupational predictors of musculoskeletal disorders among nurses [23,26]. Despite growing awareness of safe patient handling practices, the availability and utilization of mechanical lifting devices remain limited in many healthcare settings. In addition, staffing shortages may force nurses to perform patient handling activities without adequate assistance, thereby increasing ergonomic risk. These findings support international recommendations advocating the implementation of comprehensive safe patient handling programmes and greater investment in ergonomic support systems within healthcare institutions [26,32].

Another significant finding was the association between insufficient rest breaks during work shifts and severe musculoskeletal discomfort. Nurses who reported inadequate rest breaks were more than twice as likely to experience severe musculoskeletal discomfort compared with those who had sufficient opportunities for recovery. Rest breaks play a crucial role in allowing physiological recovery from repetitive physical demands and prolonged static postures. Without adequate recovery periods, cumulative muscle fatigue may develop, reducing physical resilience and increasing susceptibility to musculoskeletal injury [13,14]. Similar findings have been reported in occupational health studies demonstrating that prolonged working hours, rotating shifts, inadequate staffing, and insufficient recovery periods contribute to the development of musculoskeletal symptoms among healthcare workers [33,34].

The findings of this study have important implications for healthcare administrators, nursing managers, and policymakers. Interventions aimed at reducing musculoskeletal discomfort should extend beyond individual behavioural changes and focus on organizational and environmental modifications. Strategies such as safe patient handling programmes, increased availability of mechanical lifting equipment, ergonomic workplace redesign, adequate staffing support, structured work-rest schedules, and regular injury prevention training may help reduce musculoskeletal strain among nurses [30,34]. Given the critical role of nurses in maintaining patient safety and healthcare quality, protecting their musculoskeletal health should be regarded as an important workforce sustainability priority.

### Strengths and Limitations

This study contributes important local evidence regarding musculoskeletal discomfort among critical care nurses in a Malaysian teaching hospital, a population that has received relatively limited attention in previous Malaysian research. The use of proportionate random sampling, a high response rate, a validated musculoskeletal assessment instrument, and multivariable logistic regression analysis strengthened the methodological rigor of the study and enhanced the reliability of the findings.

Nevertheless, several limitations should be acknowledged. First, the cross-sectional design precludes causal inference between workplace exposures and musculoskeletal discomfort. Second, the use of self-reported questionnaires may introduce recall and reporting bias. Third, the study was conducted in a single teaching hospital, which may limit the generalizability of the findings to other healthcare institutions.

This study was specifically designed to examine workplace-related factors associated with musculoskeletal discomfort among critical care nurses. Consequently, the multivariable analysis focused on occupational and ergonomic exposures rather than individual characteristics. Although this approach was consistent with the study objective, unmeasured individual-level factors, such as previous musculoskeletal disorders or injuries, body mass index, comorbidities, physical activity, and psychological stress, may also influence musculoskeletal discomfort and could have acted as residual confounders. Similarly, organizational factors not evaluated in this study, including nurse-patient ratios, availability of patient-lifting equipment, and other workplace resources, may also contribute to musculoskeletal health. Therefore, residual confounding cannot be completely excluded. Future multicentre longitudinal studies should incorporate both workplace-related and individual-level factors to provide a more comprehensive understanding of musculoskeletal discomfort among critical care nurses.

## 5. Conclusions

Musculoskeletal discomfort is highly prevalent among critical care nurses working in this Malaysian teaching hospital. Manual orthopedic patient handling and insufficient rest breaks during shifts were significant predictors of severe musculoskeletal discomfort.

The findings emphasize the importance of strengthening ergonomic practices, implementing safe patient handling strategies, improving staffing support, and ensuring adequate rest periods among nurses working in critical care environments. Addressing occupational musculoskeletal risks is essential to promote workforce well-being and maintain quality patient care.

## Figures and Tables

**Table 1:** Sociodemographic characteristic of respondents (*n* = 308).

Variables	n (%)	Mean (SD)
Age		
21–30	57 (18.5)	
31–40	210 (68.2)	
41–60	41 (13.3)	
Gender		
Male	271 (88.0)	
Female	37 (12.0)	
Marital status		
Single	268 (85.4)	
Married	45 (14.6)	
Education Level		
Diploma in Nursing	299 (97.1)	
Degree in Nursing	9 (2.9)	
Weight (kg)		66.51 (15.48)
Height (m)		1.61 (0.84)
BMI		26.88 (5.54)

**Table 2:** Working characteristic and factors related to musculoskeletal discomfort among respondents (*n* = 308).

Variables	n (%)
Place of work	
Cardio Intensive Care Unit	17 (5.5)
Medical Intensive Care Unit	33 (10.7)
Surgical Intensive Care Unit	21 (6.8)
Trauma Intensive Care Unit	30 (9.7)
Medical High Dependency Care Unit	24 (7.8)
Pediatric High Dependency Unit	15 (4.9)
Neonatal Intensive Care Unit	43 (14)
Surgical High Dependency Unit	17 (5.5)
Burn Unit	11 (3.6)
Neurology Intensive Care Unit	33 (10.7)
Emergency and Trauma	64 (20.8)
Years of Services	
<5 years	87 (28.2)
6–10 years	88 (28.6)
10–15 years	76 (24.7)
>15 years	57 (18.5)
Working at the same position (standing, bending sitting)	
Yes	227 (73.7)
No	81 (26.3)
Lifting or transferring dependent patient	
Yes	275 (89.3)
No	33 (10.7)
Bending or twisting your back in an awkward way	
Yes	167 (54.2)
No	141 (45.8)
Treating an excessive number of patients on one day	
Yes	192 (62.3)
No	116 (37.7)
Carrying, lifting or moving heavy materials or equipment	
Yes	178 (57.8)
No	130 (42.2)
Performing manual orthopedic technique	
Yes	120 (39)
No	188 (61)
Not enough rest breaks or pauses during workday	
Yes	154 (50)
No	154 (50)
Work scheduling	
Yes	130 (42.2)
No	178 (57.8)
Working in awkward and cramped positions	
Yes	80 (26)
No	228 (74)
Continuing to work while injured or hurt	
Yes	137 (44.5)
No	170 (55.2)
Reaching working away from your body	
Yes	102 (33.1)
No	206 (66.9)
Anticipated sudden movement or fall by patient	
Yes	81 (26.3)
No	227 (73.7)
Inadequate training on injury prevention	
Yes	98 (31.8)
No	210 (68.2)
Working near or at physical limit	
Yes	116 (37.7)
No	192 (62.3)
Working with confuse or agitated patient	
Yes	188 (61)
No	120 (39)
Performing same task over and over	
Yes	181 (58.8)
No	127 (41.2)
Assisting patient during gait activities	
Yes	210 (68.2)
No	97 (31.5)

**Table 3:** Prevalence of musculoskeletal discomfort according to anatomical body region among critical care nurses (n = 308). Severity of musculoskeletal discomfort among respondents (n = 308).

Body Region	Frequency (n)	Percentage (%)
Lower back	235	76.3
Neck	182	59.1
Right shoulder	150	48.7
Left shoulder	136	44.2
Upper back	191	62.0
Right knee	105	34.1
Left knee	92	29.9
Right foot	157	51.0
Left foot	137	44.5
MSD Severity	Frequency (n)	Percentage (%)
Mild to moderate	55	17.9
Severe	250	82.1

**Table 4:** Logistic Regression of Working Characteristic and severe musculoskeletal discomfort among critical care nurses (*n* = 308).

Variable	Crude OR (95%CI)	*p* Value	Adjusted OR (95%CI)	*p* Value
Working at the same position (standing, bending sitting)	0.735 (0.404–1.336)	0.312		
Lifting or transferring dependent patient	0.330 (0.096–1.139)	0.079		
Manual orthopedic patient handling	1.788 (1.042–3.068)	0.035		
Treating an excessive number of patients on one day	1.114 (0.637–1.948)	0.705		
Carrying, lifting or moving heavy materials or equipment	2.019 (1.173–3.474)	0.011		
Performing manual orthopedic technique	2.019 (1.123–3.630)	0.019	1.882 (1.033–3.426)	0.039
Not enough rest breaks or pauses during workday	2.734 (1.562–4.786)	<0.001	2.581 (1.466–4.543)	<0.001
Work scheduling (overtime, irregular shifts, length of workday)	1.386 (0.798–2.406)	0.246		
Working in awkward and cramped positions	2.419 (1.240–4.720)	0.01		
Continuing to work while injured or hurt	1.841 (1.064–3.186)	0.029		
Reaching working away from your body	2.255 (1.221–4.162)	0.009		
Anticipated sudden movement or fall by patient	1.465 (0.787–2.730)	0.229		
Inadequate training on injury prevention	2.113 (1.144–3.904)	0.017		
Working near or at physical limit	1.497 (0.856–2.618)	0.158		
Working with confuse or agitated patient	0.643 (0.362–1.143)	0.132		
Performing same task over and over	0.945 (0.545–1.640)	0.841		
Assisting patient during gait activities	0.907 (0.502–1.637)	0.745		

Note: Multiple binary logistic regression analysis was performed using the enter method. Variables with *p* < 0.25 in the bivariable analysis were included in the final model. The model demonstrated good fit based on the Hosmer–Lemeshow goodness-of-fit test (*p* = 0.721), correctly classified 84.6% of cases, and showed no evidence of multicollinearity or interaction among independent variables. Statistical significance was set at *p* < 0.05.

## Data Availability

The data presented in this study are available from the corresponding author upon reasonable request. The data are not publicly available due to ethical and confidentiality restrictions.

## Abbreviations

The following abbreviations are used in this manuscript:

MSDs	Musculoskeletal Disorders
AOR	Adjusted Odd Ratio
NICU	Neonatal Intensive Care Unit
CI	Confident Interval
COR	Crude Odd Ratio
